# A First Proposal on the Nitrobenzene Photorelease Mechanism of NO_2_ and Its Relation to NO Formation through a Roaming Mechanism

**DOI:** 10.1021/acs.jpclett.3c03457

**Published:** 2024-02-19

**Authors:** Angelo Giussani, Graham A. Worth

**Affiliations:** ‡ Instituto de Ciencia Molecular, Universitat de València, Apartado 22085, ES-46071 Valencia, Spain; † Department of Chemistry, University College London, 20 Gordon Street, London WC1H 0AJ, U.K.

## Abstract

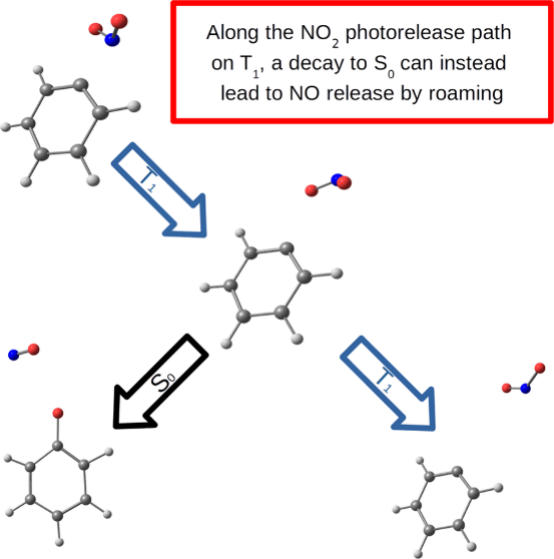

Despite the fact that NO_2_ is considered to be the main photoproduct of nitrobenzene photochemistry, no mechanism has ever been proposed to rationalize its formation. NO photorelease is instead a more studied process, probably due to its application in the drug delivery sector and the study of roaming mechanisms. In this contribution, a photoinduced mechanism accounting for the formation of NO_2_ in nitrobenzene is theorized based on CASPT2, CASSCF, and DFT electronic structure calculations and CASSCF classical dynamics. A triplet nπ* state is shown to evolve toward C–NO_2_ dissociation, being, in fact, the only low-lying excited state favoring such a deformation. Along the triplet dissociation path, the possibility to decay to the singlet ground state results in the frustration of the dissociation and in the recombination of the fragments, either back to the nitro or the nitrite isomer. The thermal decomposition of the latter to NO constitutes globally a roaming mechanism of NO formation.

Nitrobenzene has recently attracted a lot of attention in the photophysical and photochemical community.^1−7^ This is easily understandable due, from one side, to its representative role in the nitroaromatic family and, from the other side, to its peculiar photoinduced dynamics. The nitrobenzene photoresponse is in fact intrinsically key for basis science, displaying an unusual, for pure organic systems, ultrafast decay into the triplet manifold and a wide variety of photoinduced reactions. Moreover, its photochemistry has important applications in the energetic materials sector, in the study of urban atmospheric contaminants, and in the drug delivery sector.^8−10^

Nitrobenzene can photorelease NO_2_, NO, and O.^11^ The efficiency of these photoreactions is low, and no values for the quantum yields have been reported, as far as we know. It is generally considered that the formation of O is the least relevant path, while two experimental studies from Galloway et al. and Lee, Ni, and co-workers performing vacuum-ultraviolet photoionization mass spectrometry and multimass ion imaging techniques, respectively, have reported that NO_2_ is always produced in higher quantities than NO and that the formation of NO_2_ is even more favored at higher excitation energies.^11,12^ Such a vision has, however, been challenged by ultrafast electron diffraction measurements by Zewail and co-workers, who concluded that NO is instead the main result of nitrobenzene photochemistry.^13^ Despite its importance, the mechanism of NO_2_ photorelease has received very little attention, and there is no generally accepted mechanism.

The photoformation of NO has been instead the subject of various experimental and theoretical works.^12−16^ One of the most intriguing results was the determination of a bimodal distribution of the translational energy of the photoreleased NO molecules. This was first characterized by Lee, Ni, and co-workers^12^ and later reconfirmed by Suits and co-workers performing state-selected direct current slice imaging experiments.^14^ This has been recently used in the research group of Patwari in order to study the effect of different types of substituents on the photoreaction.^2,3^ Nitrobenzene can indeed photorelease NO with both high and low translational energies, and this experimental fact is taken as proof that the photoreaction can occur according to two different mechanisms. Both the study of Lee, Ni, and co-workers and the study of Suits and co-workers proposed that the fast component is formed on the T_1_ surface passing through the formation of an oxaziridine ring. Suits and co-workers also proposed that the slower NO molecules are the result of a roaming mechanism along the S_0_ surface, initially leading to the nitrite isomer. The two mechanisms can be related to the intramolecular rearrangement and dissociation-recombination mechanisms originally proposed by Chapman et. al for the photorelease of NO in nitrated polycyclic aromatic hydrocarbons (see Scheme 1).^17^ The recent work of Patwari based on the exploration of a two-dimensional model of the T_1_ potential energy surface (PES) (the C–NO_2_ and C–ONO bond distances) supports such a vision, concluding than the dynamics on the T_1_ state acts as a doorway between the roaming and nonroaming mechanisms. Performing ab initio CASSCF and CASPT2 computations and classical CASSCF dynamics, we previously put into doubt the importance of roaming in the photorelease of NO, showing how different regions of the same T_1_/S_0_ seam of intersection describing an oxaziridine ring can indeed lead to NO molecules with as much as 0.7 eV difference in their corresponding translational energy.^15^

**Scheme 1:**
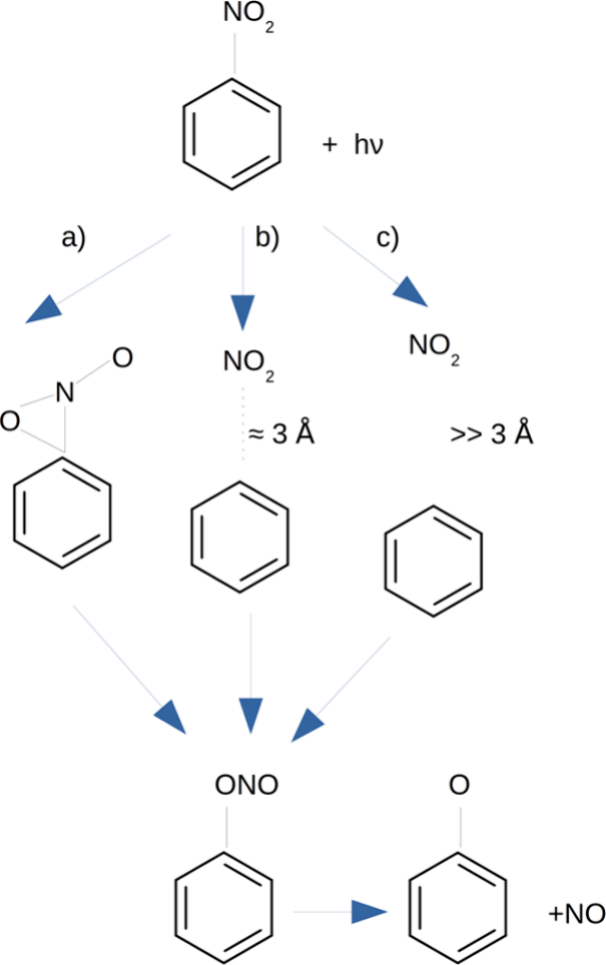
Photorelease Mechanism of NO According to (a) the Intramolecular Rearrangement Mechanism, (b) the Roaming Mechanism, and (c) the Dissociation-Recombination Mechanism

With the present contribution, we are proposing a mechanism for the photoinduced release of NO_2_ on the triplet manifold and show how along such a process a possible decay to the S_0_ surface can indeed result in the nitro-to-nitrite photoisomeriazion through a roaming mechanism. The work is based on ab initio CASSCF^18^ and CASPT2^19^ and DFT computations along with CASSCF classical dynamics simulations. Over the years, the description of the electronic structure of nitrobenzene, and of a nitroaromatics system in general, has proven to be particularly challenging.^20−25^ As active space, we employed mostly the well-tested 14 electrons in 11 orbital space, although the final results were obtained including also the sigma electrons and orbitals describing the C–NO_2_ bond, globally resulting in a CAS(16,13) space (see Figure S1).^22,23^ All wave function computations were performed with OpenMolcas,^26,27^ while DFT-B3LYP^28,29^ calculations were made with Gaussian16.^30^ Wave function calculations employed as the basis set the atomic natural orbital (ANO) of L-type contracted to C,N[4s,3p,1d]/H[2s1p],^31,32^ while the 6-311++g(d,p) basis set was used for DFT computations.^33^

As reviewed above, NO_2_ has been described as the main product of nitrobenzene photochemistry in all but one study. In a previous contribution, we suggested that such a process could be the result of the nonradiative repopulation of a hot ground state from a S_1_/S_0_ conical intersection. As this is characterized by a much shorter C–NO_2_ bond length than the S_0_ minimum (1.241 and 1.476 Å, respectively), during the evolution back to the S_0_ minimum, it can carry on along the C–NO_2_ stretching direction and eventually dissociate.^22^ Despite the plausibility of the hypothesis, CASSCF and CASPT2 classical dynamics from the mentioned S_1_/S_0_ region along the S_0_ surface, even if initially evolving toward structures having C–NO_2_ distances as long as 1.9 Å, end up in the nitrobenzene S_0_ minimum, consequently proving the tendency of the S_0_ state to evolve to the nitrobenzene structure.

Since NO_2_ formation will have to pass to C–NO_2_ dissociation, we asked ourselves which electronic state is favored (i.e., stabilized) by such a deformation, with the logical answer being the state describing a σσ* excitation of the C–NO_2_ bond. For the moment, we will focus on the triplet states. We then computed the excited state energies of the four lowest triplet states for a series of geometries obtained by systematically elongating the C–NO_2_ bond from the CASPT2 ground state minimum, where such a bond is equal to 1.47 Å, up to 2.15 Å (see Table 1, Figure S2, and Table S1). At a C–NO_2_ distance of 2.0 Å, the state describing the σσ* excitation of the C–NO_2_ bond appears among the computed states at an energy of 4.65 eV with respect to the ground state in its minimum. This indicates that the σσ* state is probably too high in energy in the ground state minimum and thermally accessible structures to be directly involved in the NO_2_ photorelease experimentally detected after 280–222 nm (4.43–5.58 eV) excitation. Regarding the other triplet states, all of them get destabilized by the C–NO_2_ elongation, although the ^3^(n_B_π*) state (following the nomenclature of ref (22), see Figure S3 of that publication) is the one that has the smallest increase in energy (see Table 1 and Figure S2). In fact, the ^3^(n_B_π*) state passes from being the T_4_ at the Franck–Condon region, to be the lowest triplet state, beside the σσ*, when the C–NO_2_ distance is equal to 2.15 Å. Looking at the n_B_ orbital (see Figures S1 and S3), we can see that it indeed partially describes a σ orbital on the C–NO_2_ bond, which consequently justifies the smaller destabilization suffer by the ^3^(n_B_π*) state.

**Table 1:** CASPT2(16,13) Energies (eV) of the Low-Lying Triplet States at the First and Last Point Computed along the C–NO_2_ Scan from the Ground State Minimum Together with Their Energy Difference (Δ*E*, eV)^a^

	energy (eV)^a^		
State^b^	C1N7 = 1.47 Å	C1N7 = 2.15 Å	Δ*E*
^3^(n_A_π*)	3.22	-	-
^3^(π_O_π*)	3.45	5.70	2.25
^3^(L_a_ππ*)	3.67	5.67	2.00
^3^(n_B_π*)	3.88	5.61	1.73
^3^(σσ*)	-	4.07	-

a All energies at all geometries composing the scan are reported in Figure S2 and Table S1. All the reported values refer to the ground state energy in its minimum.

b State nomenclature as in ref (22).

While the destabilization of some triplet states could have already been predicted from their previously published equilibrium structures,^22^ no such information was available for the ^3^(n_B_π*) state. We thus performed a CASSCF(14,11) optimization of this state, obtaining a minimum characterized by a C–NO_2_ distance of 1.449 Å. Since dynamic correlation has already proved to be key in the determination of reliable structural parameters for the ground state minimum of nitrobenzene, the CASSCF(14,11) ^3^(n_B_π*) minimum was numerically reoptimized at the CASPT2(14,11) level. The resulting structure, hereafter ^3^(n_B_π*)_min-caspt2_, is actually the first and only point obtained along a numerical CASPT2(14,11) minimum energy path (MEP) from the CASSCF(14,11) minimum, from which the further evolution of the ^3^(n_B_π*) state we were unable to describe at the same level of theory. The ^3^(n_B_π*)_min-caspt2_ structure is however characterized by a single numerical CASPT2 imaginary frequency of 47 cm^–1^ and can consequently be indeed considered as a CASPT2 minimum but constrained on the MEP hypersphere. More importantly, at the ^3^(n_B_π*)_min-caspt2_ geometry, the C–NO_2_ distance is equal to 1.497 Å, which is indeed the largest C–NO_2_ distance of all previously characterized minima, both singlet and triplet. Additionally, the nitro group is significantly rotated with respect to the plane of the ring and describes a substantial pyramidalization (see Figure 1). Regarding its energy position, at the ^3^(n_B_π*)_min-caspt2_ structure the ^3^(n_B_π*) state is the T_3_ state, 0.27 and 0.36 eV above the T_2_ and T_1_ states, respectively.

**Figure 1:**
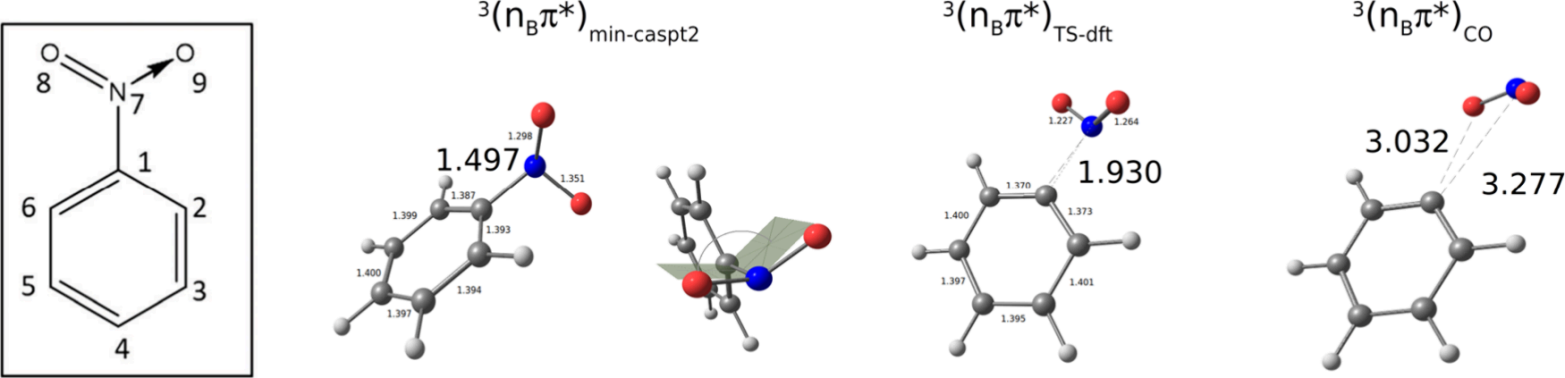
Key geometries of nitrobenzene. Bond lengths are reported in Å. In the inset are displayed the nitrobenzene structure and atom labeling.

In order to further study the ^3^(n_B_π*) state along the C–NO_2_ stretching coordinate, we resort to using DFT calculations and ran a series of transition state (TS) optimizations on the triplet manifold starting from geometries displaying a significant elongation of the C–NO_2_ bond. A B3LYP DFT transition state on the T_1_ PES describing the dissociation of the NO_2_ bond was obtained, hereafter ^3^(n_B_π*)_TS-dft_ (see Figure 1). The only imaginary frequency characterizing this structure is equal to 463 cm^–1^ and indeed describes the NO_2_ dissociation (see Figure S4). From the analysis of the corresponding DFT electron density and orbitals and performing a TDDFT computation, the T_1_ state at the characterized TS geometry has indeed a marked ^3^(n_B_π*) character (see Figure S5). Different attempts to obtain such TS at the CASSCF and CASPT2 level were unsuccessful.

The CASSCF and CASPT2 energies at the ^3^(n_B_π*)_TS-dft_ structure were computed enlarging the active space to 16 electrons in 13 orbitals, to account for the σσ* system of the breaking C–NO_2_ bond (see Figure S1). The use of such an enlarged (16,13) active space is also supported by previous computations on the dissociation process.^22,23^ At both the CASSCF(16,13) and CASPT2(16,13) level, the ^3^(n_B_π*) state is the lowest triplet state at the ^3^(n_B_π*)_TS-dft_ structure, while at the ^3^(n_B_π*)_min-caspt2_ geometry, it is the T_3_ state. Despite that, the CASPT2(16,13) energy of the ^3^(n_B_π*) state is 0.27 eV lower in energy at the ^3^(n_B_π*)_min-caspt2_ structure with respect to the ^3^(n_B_π*)_TS-dft_ geometry, in agreement with the minimum nature of the former point. In order to better evaluate the PES separating the ^3^(n_B_π*)_min-caspt2_ and ^3^(n_B_π*)_TS-dft_ points, a CASPT2(16,13) LIIC calculation connecting the two structures was performed, resulting in an energy barrier for the evolution from the former to the latter of 0.97 eV (see Figure S6).

From the ^3^(n_B_π*)_TS-dft_, the subsequent evolution of the ^3^(n_B_π*) state was characterized by both performing CASSCF(14,11) MEP calculations and running CASSCF(14,11) dynamics (energy along the trajectory shown in Figure S7). In both cases, the system evolves toward complete NO_2_ dissociation, so we can conclude that the population of the ^3^(n_B_π*) state, the decay to its corresponding minimum, and the subsequent evolution to the ^3^(n_B_π*)_TS-dft_ structure surmounting the upper bound energy barrier of 0.97 eV, constitutes a plausible mechanism for the photoinduced formation of NO_2_ in nitrobenzene.

A frustrated NO_2_ dissociation would be part of the roaming mechanism for the nitro-to-nitrite photoisomerization. We then ask ourselves if somehow along the described NO_2_ photorelease process the system could instead follow a nondissociative path toward the formation of the nitrite species. Looking at the CASSCF(14,11) dynamics on the T_1_ surface from the ^3^(n_B_π*)_TS-dft_ structure, it is possible to observe that the dissociating NO_2_ fragment rotates in such a way that the orientation of the nitro group with respect to the aromatic ring is inverted. That means that along the dissociation path the system describes a series of geometries for which the oxygen atoms are closer to the carbon atom of the aromatic ring previously attached to the nitrogen atom than the nitrogen atom itself. The phenomenon is displayed in Figure 2, together with a representative geometry, hereafter ^3^(n_B_π*)_CO_, where indeed the C1O8 distance is significantly smaller than the C1N7 (3.032 and 3.277 Å, respectively, see Figure 1). These values are however large enough to describe a predissociation status, and indeed, in the ^3^(n_B_π*)_CO_ structure, the T_1_ and S_0_ states, both describing an unpaired electron on each fragment, are very close in energy (0.27 eV). Such a situation will, in principle, allow transfer of population from T_1_ to S_0_, although the two display a low spin–orbit coupling of 0.75 cm^–1^ as a result of their common nature. From the latter, CASSCF(14,11) dynamics on the S_0_ surface display indeed a peculiar behavior. At first, the system neither continues toward dissociation nor returns to a single bonded structure, while after around 150 fs, it abruptly decays toward nitrite formation (see Figure S8). We can then hypothesize that the key event that determines if the system will either photorelease NO_2_ or photoisomerize is the passage from the T_1_ to the S_0_ state along the NO_2_ dissociation process. It is also important to note when such a T_1_ to S_0_ decay takes place, since in the case where population transfer occurs at a geometry characterized by a shorter C1N7 than C1O distance, the ground state will actually return to the initial nitrobenzene structure, then globally describing a nonphotoreactive decay.

**Figure 2:**
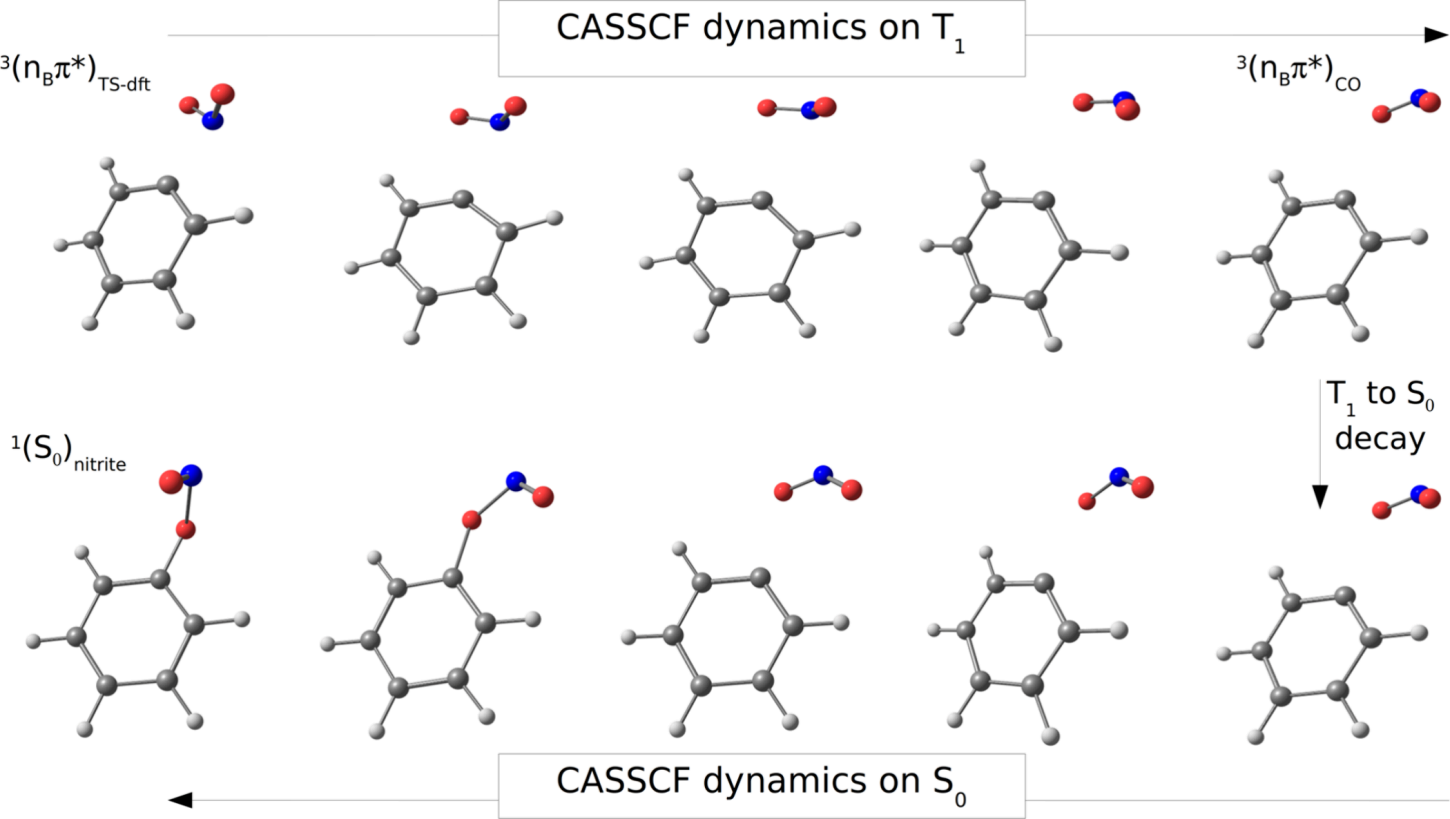
Geometrical evolution along the T_1_ surface from the ^3^(n_B_π*)_TS-dft_ to the ^3^(n_B_π*)_CO_ structure, and subsequent evolution from the latter structure along the S_0_ surface toward the nitrite isomer.

In summary, we have here characterized a possible mechanism of the photorelease of NO_2_ in nitrobenzene. The process occurs on the triplet manifold, and the key protagonist is the triplet ^3^(n_B_π*), which is the state among the accessible low-lying excited states that is least destabilized by the C–NO_2_ stretching. From its minima, already characterized by a relatively large C–NO_2_ bond length, the system can further evolve toward NO_2_ dissociation, surmounting an energy barrier of at most 0.97 eV. Along the dissociation process, a combination of the reorientation undergone by the NO_2_ fragment together with the approach of the complete dissociation limit where T_1_ and S_0_ are degenerate allows the decay from the former to the latter state, which instead of dissociating tends to react back forming either the nitrite or the nitro isomer. In the former case, the subsequent thermal release of NO will complete the roaming photorelease of NO in nitrobenzene, actually being the photoactivated and roaming part of the process the formation of the nitrite isomer.

Additionally, we attempted to evaluate whether a similar process could also occur entirely along the singlet manifold through the singlet ^1^(n_B_π*) state and obtained that much higher energies are required and that the S_0_ always tends toward a bonded structure.

At this point, it is worth putting into comparison the current results with the previous picture. Figure 3 is an attempt in this direction, showing the emerging global photochemical landscape of nitrobenzene based on current and previous outcomes (see also Figure S9 where the energies are given in kcal/mol).^15^ On the right side of Figure 3, the newly described paths leading to NO_2_ formation are depicted along with the related roaming mechanism, which results first in the nitrite isomer and then the release of NO. The left side of Figure 3 reports the previously characterized paths passing from the T_1_/S_0_ oxaziridine-like singlet triplet crossing regions and leading respectively to the direct release of NO and to the production of NO after first forming an epoxide structure. The previously characterized roaming-like path is not reported, now replaced by the new roaming mechanism described here.

**Figure 3:**
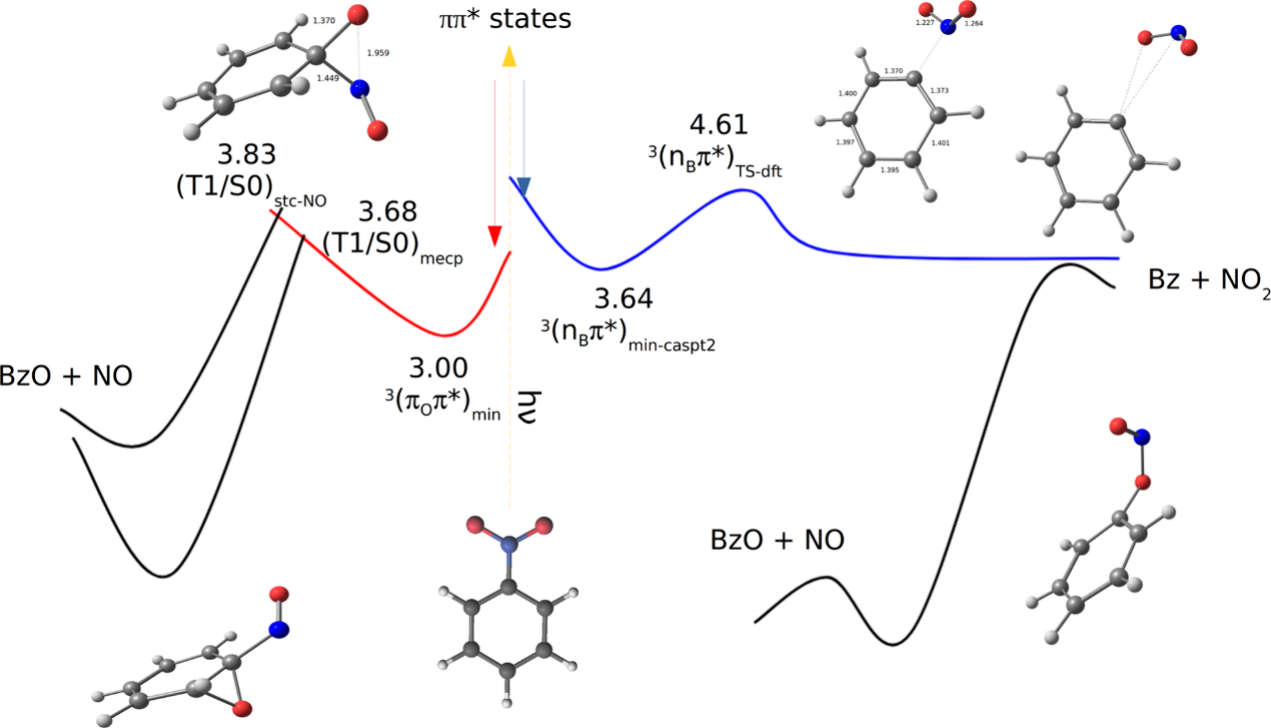
Schematic representation of the main photochemical routes for nitrobenzene. All the reported CASTP2(16,13) energies (eV) refer to the ground state energy at its minimum. On the right side, the here-described new paths are depicted, while on the left side the paths described in ref (15) are presented.

Finally, it is always important to keep in mind the limitations of the proposed model. Some of the results are based on a single zero-velocity CASSCF classical dynamics simulation. Although in the present contribution we are not aiming at quantitative results, still drawing a conclusion on the basis of a single trajectory can be misleading, since it would not be able to capture possible significant dynamics effects that only an ensemble of trajectories will be able to show. We then decided to reproduce the results using a purely static approach, computing MEPs. In particular, a MEP calculation starting from the ^3^(n_B_π*)_TS-dft_ geometry on the T_1_ surface has been performed, which, similarly to the corresponding dynamics, evolves toward NO_2_ formation, although describing a less pronounced rotation of the NO_2_ fragment. At the ninth point of the MEP, hereafter ^3^(n_B_π*)_CO-mep_, the C1N7 and C1O8 distances are equal to 3.018 and 3.031 Å, respectively, and the energy gap between S_0_ and T_1_ is equal to 0.09 eV. This energy difference will even better justify a possible T_1_ to S_0_ decay than the ones characterizing the ^3^(n_B_π*)_CO_ geometry (0.27 eV). From this MEP point, the computation of both CASSCF(14,11) dynamics and MEP on the S_0_ surfaces ended in the nitrite isomer, consequently supporting the proposed mechanism on the basis of static calculations.

## Supporting Information Available

Active space orbitals, CASPT2(16,13) energies of the lowest triplet states, description of the nature of the ^3^(n_B_π*) state, imaginary frequency at the TS, CASSCF(14,11) energies of T_1_ and S_0_ along the performed dynamics, schematic representation of the main photochemical routes (using kcal/mol), and Cartesian coordinates of the optimized points (PDF)
Transparent Peer Review report available (PDF)
