# Supplementary material for: A First Proposal on the Nitrobenzene Photorelease Mechanism of NO2 and Its Relation to NO Formation through a Roaming Mechanism
Source: J Phys Chem Lett. 2024 Feb 19;15(8):2216–21. doi: 10.1021/acs.jpclett.3c03457 (PMC10910573; doi:10.1021/acs.jpclett.3c03457)
Supplement: Supplementary file 1 — jz3c03457_si_001.pdf [file jz3c03457_si_001.pdf]

Supporting Information
(Total of 8 pages)
for

A First Proposal on Nitrobenzene Photorelease Mechanism of NO_2, and its Relation to NO Formation Through Roaming Mechanism

Angelo Giussani[*,‡] and Graham A. Worth[†]

[‡]Instituto de Ciencia Molecular, Universitat de València, Apartado22085, ES-46071 Valencia, Spain
[†]Department of Chemistry, University College London, 20 Gordon Street, London WC1H 0AJ, U.K.
*To whom correspondence should be addressed. Email: Angelo.Giussani@uv.es

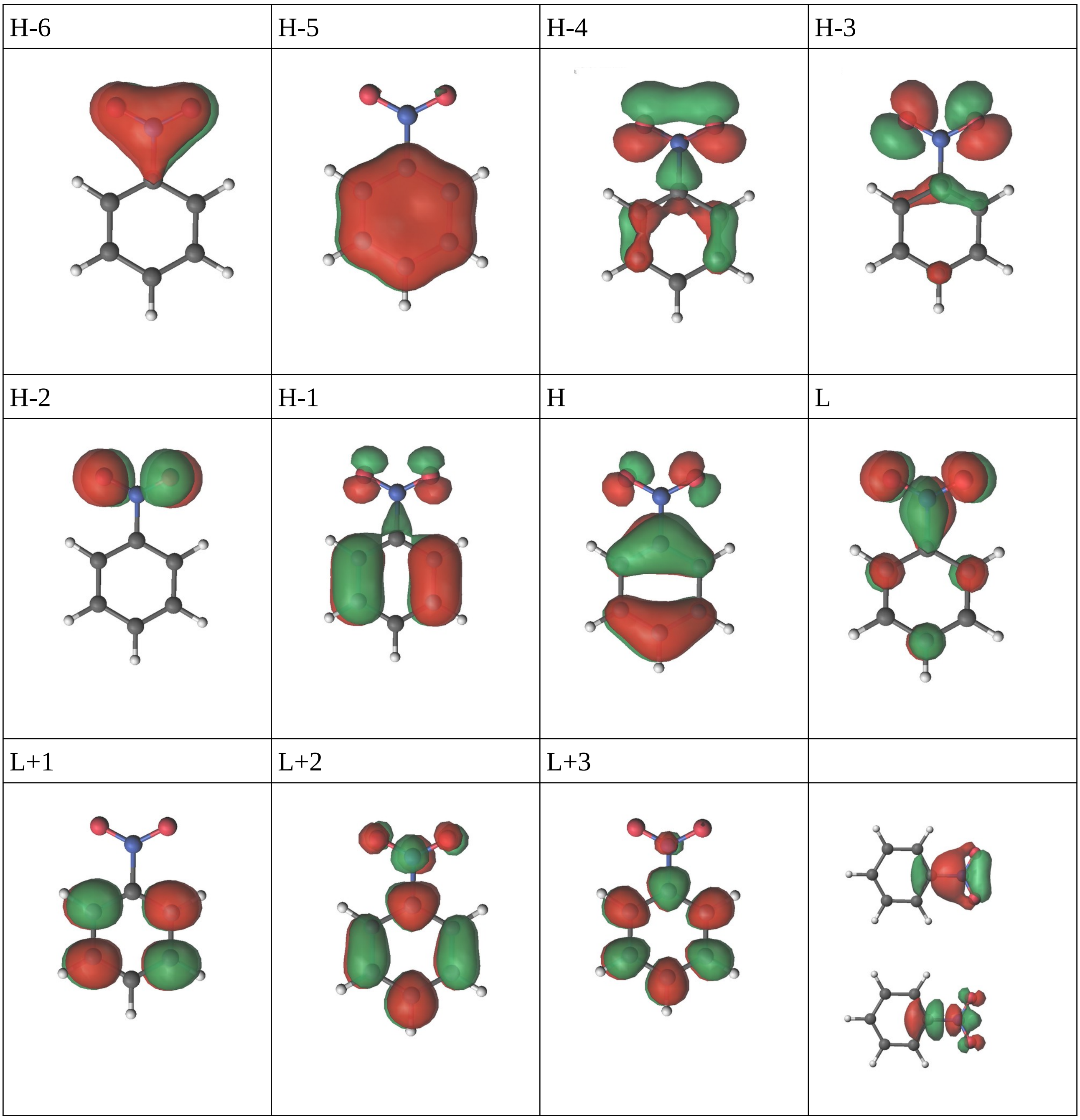

Figure S1. CAS(16,13) active space of nitrobenzene. The CAS(14,11) active space results from the exclusion of the σ and σ* orbitals display in the bottom left corner.

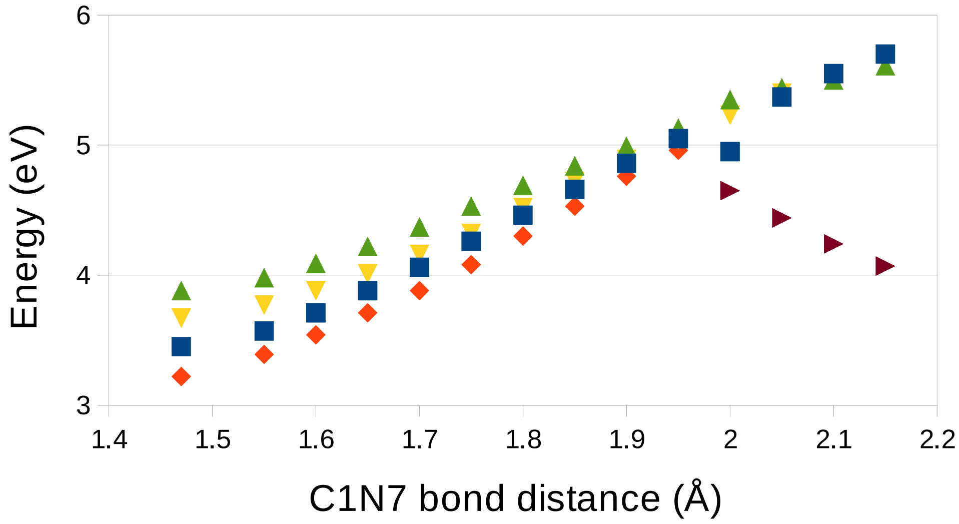

Figure S2. CASPT2(16,13) energies (eV) of the low-lying triplet states along the C-NO_2 stretching coordinates from the ground state minimum. Green triangles, yellow triangles, blue squares, red diamonds, and purple triangles represent the energies of the $^3(n_B\pi^*)$, $^3(L_a\pi\pi^*)$, $^3(\pi_O\pi^*)$, $^3(n_A\pi^*)$, $^3(\sigma\sigma^*)$ states (following the nomenclature of ref. 22), respectively. All the reported values refer to the ground state energy in its minimum.

Table S1. CASPT2(16,13) energies (eV) of the low-lying triplet states (following the nomenclature of ref. 22) along the C-NO_2 stretching coordinates from the ground state minimum. All the reported values refer to the ground state energy in its minimum.

	state				
C1N7 bond (Å)	$^3(n_A\pi^*)$	$^3(\pi_O\pi^*)$	$^3(L_a\pi\pi^*)$	$^3(n_B\pi^*)$	$^3(\sigma\sigma^*)$
1.47	3.22	3.45	3.67	3.88	-
1.55	3.39	3.57	3.77	3.98	-
1.60	3.54	3.71	3.88	4.09	-
1.65	3.71	3.88	4.01	4.22	-
1.70	3.88	4.06	4.16	4.37	-
1.75	4.08	4.26	4.32	4.53	-
1.80	4.30	4.46	4.52	4.69	-
1.85	4.53	4.66	4.72	4.84	-
1.90	4.76	4.86	4.89	4.99	-
1.95	4.96	5.05	5.04	5.13	-
2.00	-	4.95	5.23	5.35	4.65
2.05	-	5.37	5.40	5.44	4.44
2.10	-	5.55	5.54	5.50	4.24
2.15	-	5.70	5.67	5.61	4.07

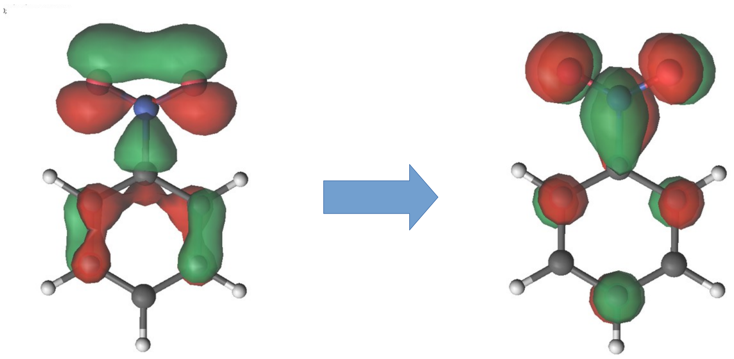

Figure S3. Orbitals involved in the one-electron promotion mainly describing the $^{3}(n_B\pi^*)$ state

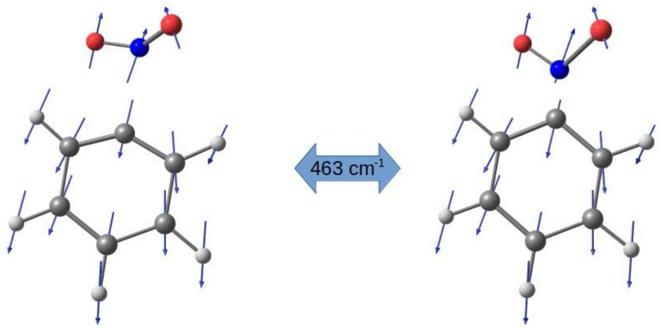

Figure S4. B3LYP imaginary frequency characterizing the $(n_B\pi^*)_{TS\text{-}dft}$ TS.

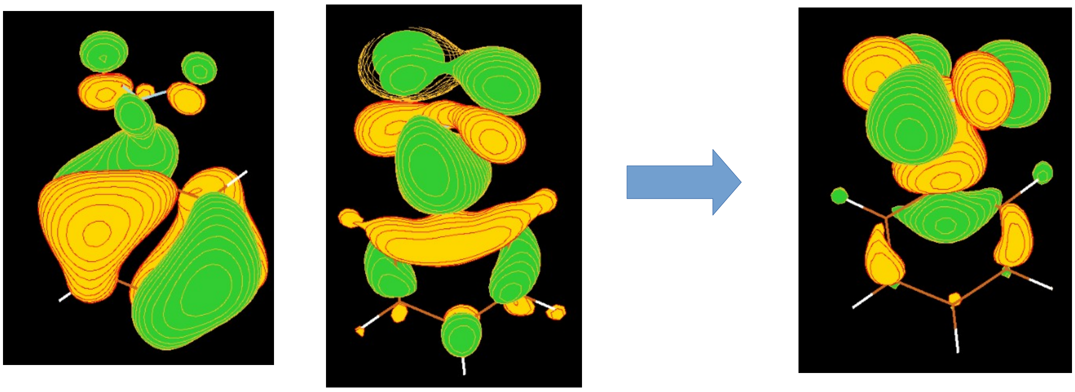

Figure S5. Main orbitals describing the T1 state at the .$(n_B\pi^*)_{TS\text{-}dft}$ geometry at the TDDFT level.

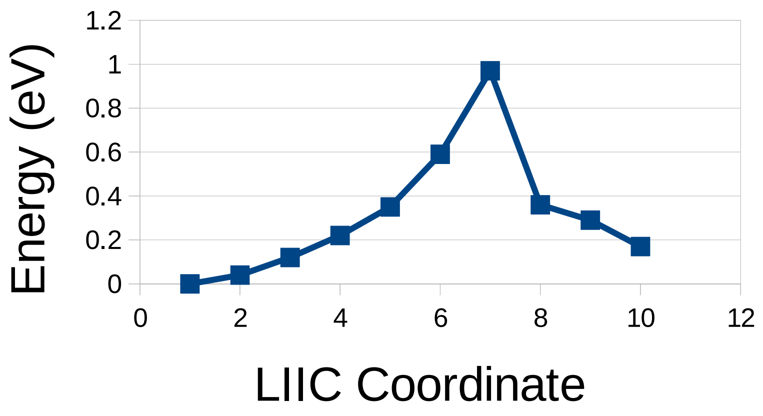

Figure S6. CASPT2(16,13) energies (eV) of the $^3(n_B\pi^*)$ state along the LIIC path connecting the $^3(n_B\pi^*)_{min\text{-}caspt2}$ and $^3(n_B\pi^*)_{TS\text{-}dft}$ structures (represented as the first and last point in the graphic, respectively). All the reported values refer to the $^3(n_B\pi^*)$ energy in its minimum, $^3(n_B\pi^*)_{min\text{-}caspt2}$.

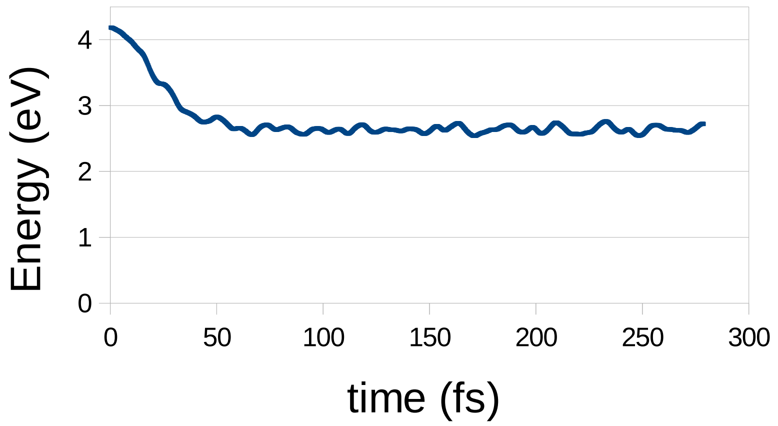

Figure S7. Evolution of the T_1 CASSCF(14,11) energy (eV) along the dynamics on the T_1 state from the $^3(n_B\pi^*)_{TS\text{-}dft}$ structure.

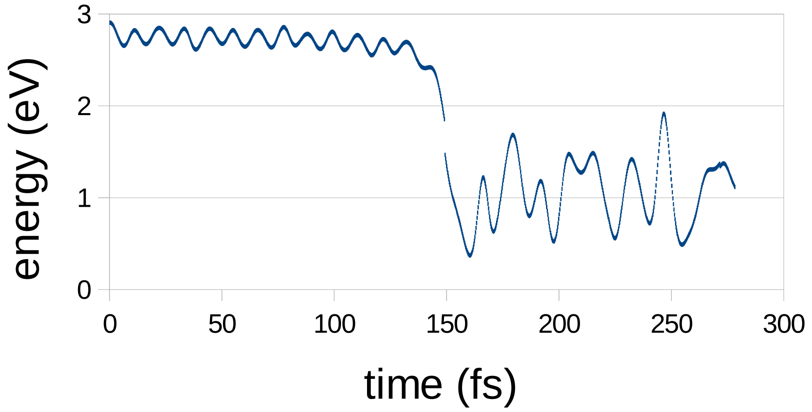

Figure S8. Evolution of the S_0 CASSCF(14,11) energy (eV) along the dynamics on the S_0 state from the $^3(n_B\pi^*)_{CO}$ structure.

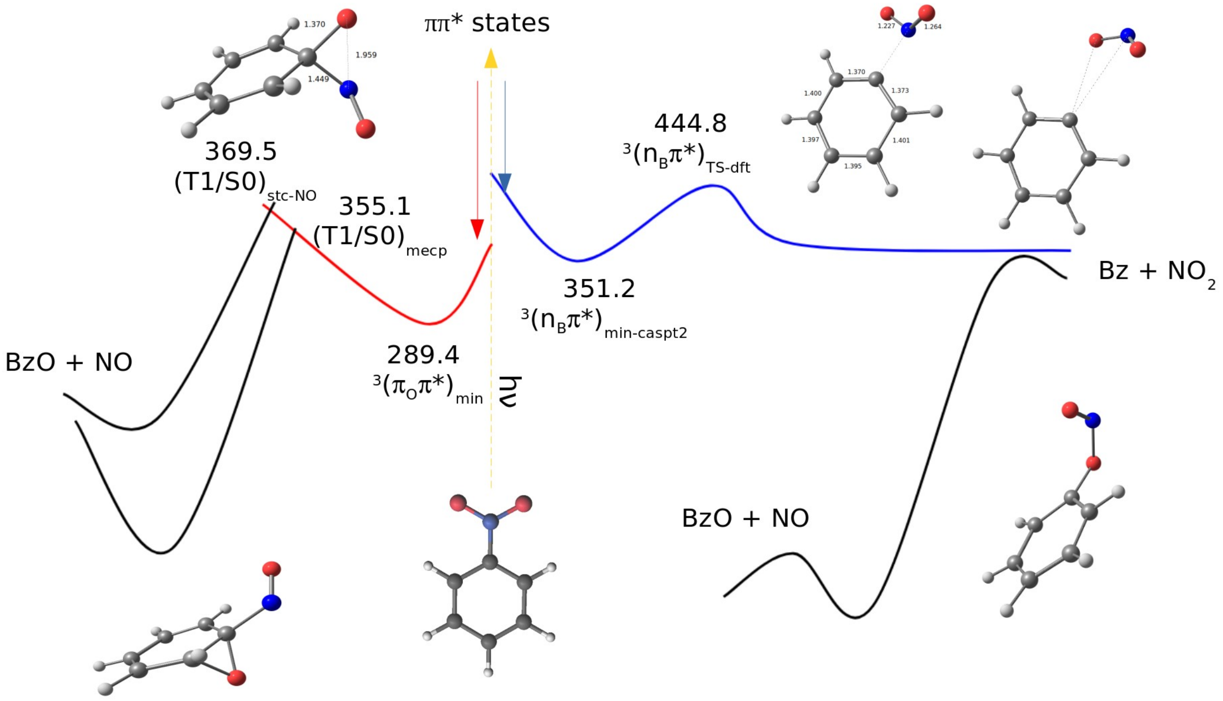

Figure S9. Schematic representation of the main photochemical routes for nitrobenzene. All the reported CASPT2(16,13) energies (kcal/mol) refer to the ground state energy at its minimum. On the right side, the here described new paths are depicted, while on the left side the path described in reference 15 are presented.

Table S2. Cartesian coordinates x, y, z (in Å) of the characterized key geometries of nitrobenzene.

$^3(n_B\pi^*)_{min\text{-}casscf}$

H	1.163606	-1.940384	-0.040408
C	0.659946	-1.118469	-0.511804
C	0.551155	-1.035422	-1.898248
C	-0.100080	0.027735	-2.479346
C	-0.656890	1.030528	-1.687482
C	-0.558012	0.962555	-0.306881
C	0.105492	-0.119599	0.266023
H	0.978949	-1.807383	-2.510947
H	-0.181489	0.087549	-3.549225
H	-1.164746	1.858487	-2.146359
H	-0.982779	1.726600	0.315827
N	0.241620	-0.243931	1.703531
O	0.857919	0.817325	2.211025
O	-1.051417	-0.409215	2.247465

$^3(n_B\pi^*)_{min\text{-}caspt2}$

C	0.665650	-1.130704	-0.503736
C	0.098324	-0.119772	0.258180
C	-0.563286	0.970763	-0.301034
C	-0.660274	1.036986	-1.690576
C	-0.104262	0.034663	-2.489652
C	0.558902	-1.046738	-1.896562
N	0.202211	-0.199169	1.749415
O	0.885814	0.808358	2.198326
O	-1.040006	-0.437044	2.223898
H	-1.175030	1.877266	-2.150047
H	-0.989685	1.737031	0.337929
H	-0.182816	0.090346	-3.572599
H	0.992884	-1.826383	-2.517627
H	1.172547	-1.960287	-0.022179

$^3(n_B\pi^*)_{dft\text{-}ts}$

C	-0.554445	-1.280502	-0.053389
C	0.135042	-0.095512	0.012088
C	-0.384503	1.171216	0.073097
C	-1.781200	1.253038	0.030840
C	-2.552826	0.091859	-0.056194
C	-1.949781	-1.165206	-0.102956
N	2.046751	-0.166543	-0.240113
O	2.312299	-0.972020	0.696640
O	2.469299	0.969667	-0.430396
H	-2.258176	2.226589	0.057400
H	0.251944	2.046870	0.118387
H	-3.633370	0.168986	-0.085927
H	-2.555982	-2.062008	-0.165734
H	-0.053932	-2.239672	-0.065622

$^3(n_B\pi^*)_{CO}$

C	-0.86296076	-1.21472081	0.00983261
C	-0.12987122	-0.06960552	0.10546802
C	-0.72052346	1.16374426	0.12899762
C	-2.09871443	1.23094735	0.01346206
C	-2.88660633	0.10599212	-0.09534907
C	-2.23757300	-1.10093330	-0.11064044
N	3.13715089	-0.17480817	-0.13521722
O	2.63497711	-1.00429036	0.56812579
O	2.67822491	0.89707531	-0.50423894
H	-2.54490161	2.21435687	0.01731434
H	-0.12938430	2.06054735	0.22635394
H	-3.95956898	0.17184940	-0.18695545
H	-2.80088026	-2.01616472	-0.21466649

H	-0.39191656	-2.18402789	0.00495180

$^3(n_B\pi^*)_{CO\text{-}mep}$

C	-0.837005	-1.247792	-0.020557
C	-0.134455	-0.068873	0.075206
C	-0.689691	1.193416	0.093345
C	-2.077712	1.265064	0.011867
C	-2.830678	0.097806	-0.086325
C	-2.225719	-1.142521	-0.104157
N	2.860159	-0.235668	-0.264698
O	2.704922	-0.964116	0.644731
O	2.738641	0.925019	-0.404903
H	-2.569659	2.220562	0.023942
H	-0.083718	2.076100	0.167523
H	-3.901422	0.165138	-0.149148
H	-2.826230	-2.030873	-0.180712
H	-0.344661	-2.201665	-0.026851
