# Supplementary material for: A First Proposal on the Nitrobenzene Photorelease Mechanism of NO2 and Its Relation to NO Formation through a Roaming Mechanism
Source: J Phys Chem Lett. 2024 Feb 19;15(8):2216–21. doi: 10.1021/acs.jpclett.3c03457 (PMC10910573; doi:10.1021/acs.jpclett.3c03457)
Supplement: Supplementary file 2 — jz3c03457_si_002.pdf [file jz3c03457_si_002.pdf]

jz-2023-03457f.R1

Name: Peer Review Information for "A First Proposal on the Nitrobenzene Photorelease Mechanism of NO₂, and Its Relation to NO Formation Through a Roaming Mechanism"

First Round of Reviewer Comments

Reviewer: 1

Comments to the Author

Comments: The present manuscript reports a photoinduced mechanism that accounts for the formation of NO2 in nitrobenzene and its subsequent thermal decomposition to NO at triplet state. Unfortunately, although the current manuscript appears to contain considerable computational work, it does not meet the high standards required by JPCL.

1. The authors stated “The efficiency of these photoreactions is low, and no quantum yields have, as far as we known, ever been reported” in early work of Ref. 11-13. This statement is somewhat one-sided and misleading for general readers. In fact, roaming-related researchers have conducted quantum yield in Ref. 14, and the authors perhaps also performed some studies [Phys. Chem. Chem. Phys., 2019, 21, 10514-10522; Phys. Chem. Chem. Phys., 2020, 22, 15945-15952].

2. According to Suits and co-workers in Ref. 14, “Instead, the fast component is probably due to dissociation on the excited triplet state, as suggested by Ni and co-workers”. Therefore, the importance of Ni and co-workers should be also acknowledged in “Suits and co-workers proposed that the fast component is formed on the T1 surface”.

3. In Scheme 1, the authors demonstrated the two mechanisms on the S0 and T1 surfaces, which are related to the work proposed by Chapman et. al. However, the important roaming reaction path at S0 surface is not depicted. Please complement the Scheme 1 and related descriptions.

4. Following the authors’ emphasis, their previous outcomes have been carefully read. It was found that left paths in Figure 3 have been observed [Phys. Chem. Chem. Phys., 2020, 22, 15945-15952]. The values of 3.83 eV and 3.68 eV are consistent with previous work, and the value of 3.00 eV is similar to the previously reported 3.04 eV, but these values are not discussed in the manuscript. Furthermore, it is recommended to label the roaming reaction path (i.e., path 3 from previous work) as well as the symbols (i.e., (T1/S0)mecp and (T1/S0)stc- from previous work) in Figure 3. Finally, it is crucial to appropriately cite and discuss results that are consistent with prior studies in both the legend and discussions.

5. The authors presented the non-roaming reaction pathway on both the S0 and T1 surfaces starting from 3(nB[illegible]*)CO in Figure 2 and the evolution of the S0 CASSCF(14,11) energy along the dynamics on the S0 state in Figure S6. On the one hand, it is recommended that the authors include information about the evolution of the T1 CASSCF(14,11) energy and describe related details, such as time step, based on the work [Nat. Chem., 14, 914-919].

6. Although the title mentions "nitrobenzene photorelease mechanism of NO2 and its relation to NO formation through a roaming mechanism", such relation is indeed lacking. Throughout the entire manuscript, there is a predominant focus on the non-roaming reaction pathway. Please reassess the significance of this work.

7. Table 1 shows CASPT2(16,13) energies at the first and last point along the C-NO2 scan from the ground state minimum. The authors should calculate CASPT2(16,13) energies for each state throughout the entire scanning process, which may bring more intriguing results.

8. The author mentioned “In fact, the 3(nB[illegible]*) state passes from being the T4 at the Frack-Condon region, to be the lowest triplet state, beside the [illegible]*, when the C-NO2 distance is equal to 2.15 Å.” However, the result of T4 state is not clarified. Please cite the related reference or include further discussions.

9. The author mentioned “electron density and perform TDDFT computation”. However, the manuscript lacks a clear description of this aspect. Please include further discussions.

10. For the roaming reaction pathway, the authors should calculate quasi-classical dynamics or special post-TS dynamics, rather than CASSCF classical dynamics.

Minor:

1. The authors used eV as the energy unit, which is seemingly popular in condensed-phase, but much less in molecular chemistry nor the previous roaming studies. This issue is not critical.

Reviewer: 2

Comments to the Author

The manuscript by Giussani and Worth describes the photorelease of NO as a consequence of frustrated C–N bond dissociation leading to the formation of NO2 in nitrobenzene. In an earlier contribution [Ref. 15] they had shown that the roaming pathway for the NO release proceeds via a high energy (5.13 eV) T1/S0 STC, which restricts its general applicability. Based on the some of the recent literature {Refs. 2-5] the authors have revisited this problem and have shown that roaming mechanism can indeed proceed at much lower energy (4.41 eV). Authors have shown that the NO2

fragment undergoes reorientational motion around the singlet-triplet degeneracy near the dissociation limit allows the system to revert to a nitro or nitrite isomer upon decay into the singlet ground state. This is a timely contribution to understand the origin of the roaming mechanism in the nitro to nitrite isomerization pathway and subsequent NO release.

Major issues

1. The opening paragraph needs to be rephrased in terms of the motivation for the present work, the photochemistry of nitrobenzene is anything but prototypical, since there has been a lot of effort both experimentally and theoretically to address the complicated dynamics. Repetitive use of photophysical and photochemical words.

2. For calculating NO2 release from nitrobenzene, the author has considered the contribution from four excited triplet states even though primary excitation should be in some highly excited singlet states. Therefore, the motivation behind restricting the calculation between triplet states must be clearly be bought out.

3. The author has shown the NO2 release by stretching the C-N bond from 1.47 to 2.15 Å in the CASPT2 level. But nitro-nitrite photoisomerization through roaming dynamics occurs through C-N distance greater than 3 Å. Is the bond length stretching up to 2.15 Å enough to capture the actual dynamics?

4. On page number 3 and lines number 15-20, the author mentioned “At the C-NO2 distance of 2.0 Å, the state describing the σσ* excitation of the C-NO2 bond appears among the computed states, at an energy of 4.65 eV with respect to the ground state in its minimum. This indicates that the σσ* state is probably to high in energy to be directly involved in the NO2 photo release experimentally detected after 280-222 nm (4.43-5.58 eV) excitation.” Probability of contribution from σσ*state at C-NO2 distance of 2.0 Å should be higher in between excitation range 4.43-5.58 eV. These sentences are confusing, needs better paraphrasing. Also “to high” should be “too high”.

5. Authors have proposed a mechanism for the photoinduced release of NO2 from nitrobenzene from the triplet manifold whereas the singlet-triplet crossing leads to either the nitrite or nitro isomer and resulting in NO release. What is the magnitude of the singlet-triplet coupling constant at this crossing point?

6. In nitroaromatic compounds, the spin-orbit coupling and singlet-triplet crossing are prominent excited state processes, and fluorescence observed in them is negligible, therefore the formation of NO2 fragment can be associated with the ground state channel. Do the authors suggest that NO2 release is not a possible channel from the S0 state as however long the C-NO2 bond is elongated?

Minor issues

7. On page number 1 and line number 48, please correct the spelling of Zewail for reference 13.

8. In Figure 3 right panel, the notation of the end product should be Bz + NO2

9. Scheme 1 a) the word “intermolecular rearrangement “should be changed with “intramolecular rearrangement”.

10. Reference 4 is not properly sighted.

11. Page 1 Line 25: ‘Singlet’ ground state instead of ‘single’ ground state

12. Page 5 Line 3: The term ‘orientation of nitro group with respect to the aromatic ring is flipped’ does not seem accurate here. The rotational reorientation indeed occurs leading to the oxygen atom being closer to the carbon, but the term ‘flip’ may not be the correct usage.

13. Page 2 Line 8: While discussing the work of Patwari, it may be ideal to mention that the work looks at the PES along the T1 surface and the dynamics on the triplet surface acts as a doorway between the roaming and non-roaming mechanisms.

14. Page 1 Line 44: ‘produced’ instead of ‘produce’

Author's Response to Peer Review Comments:

Valencia, January 14th, 2024

Dear Prof. Editor,

Please find attached the revised version of our paper entitled: “A first proposal on nitrobenzene photorelease mechanism of NO_2, and its relation to NO formation through roaming mechanism”, by Angelo Giussani and Graham Worth.

We thank you and the referees for your efforts. In separate pages we list our comments to the referees’ suggestions and the changes introduced in the manuscript. We hope that the current revised version will be suited for publication in The Journal of Physical Chemistry Letter.

With our best regards,
Dr. Angelo Giussani

Comments and changes after Referee 1

Main concerns:

1- The referee states: "The authors stated “The efficiency of these photoreactions is low, and noquantum yields have, as far as we known, ever been reported” in early work of Ref. 11-13. This statement is somewhat one-sided and misleading for general readers. In fact, roaming-related researchers have conducted quantum yield in Ref. 14 ... "

No quantitative values of the quantum yields of the photoreactions are reported in the literature. The mentioned sentence at the end of page 1 has now been slightly modified as follows: "*The efficiency of these photoreactions is low, and no values for the quantum yields have, as far as we know, ever been reported ...*"

2- The referee states: "...the importance of Ni and co-workers should be also acknowledged in“Suits and co-workers proposed that the fast component is formed on the T1 surface”."

Indeed Ni and co-workers proposed that the fast component is formed on the lowest triplet potential energy surface. Consequently, the original sentence "*Suits and co-workers proposed that the fast component is formed on the T1 surface passing through the formation of an oxaziridine ring, while a roaming mechanism along the S0 and initially leading to the nitrite isomer should account for the slower molecules.*" has now been replaced (page 2) with "*Both the study of Lee, Ni and co-workers and the study of Suits and co-workers proposed that the fast component is formed on the T_1 surface passing through the formation of an oxaziridine ring. Suits and co-workers also proposed that the slower NO molecules are the result of a roaming mechanism along the S_0 surface initially leading to the nitrite isomer.*"

3- The referee states: "In Scheme 1, the authors demonstrated the two mechanisms on the S0 and T1surfaces, which are related to the work proposed by Chapman et. al. However, the important roaming reaction path at S0 surface is not depicted. Please complement the Scheme 1 and related descriptions."

The idea was to relate the roaming mechanism with the dissociation-recombination mechanism proposed by Chapman et. al. Hovewer, it is true that the two can be different, the first describing a frustrate dissociation, the second a proper dissociation, so, following the referee suggestion we have now include in Scheme 1 the roaming mechanism.

4- The referee states: "...it is recommended to label the roaming reaction path (i.e., path 3 fromprevious work) as well as the symbols (i.e., (T1/S0)mecp and (T1/S0)stc- from previous work) in Figure 3. Finally, it is crucial to appropriately cite and discuss results that are consistent with prior studies in both the legend and discussions."

The mentioned symbols (i.e.: (T1/S0)mecp and (T1/S0)stc-NO) are now included in Figure 3. In order to better discuss the new results in comparison with previous outcomes, the following paragraph has been added on page 6 of the revised text: "*On the right side of Figure 3, the newly described paths leading to NO_2 formation are depicted along with the related roaming mechanism which results firstly in the nitrite isomer and then the release of NO. The left side of Figure 3 reports the previously characterized paths passing from the T_1/S_0 oxaziridine-like singlet triplet crossing regions and leading respectively to the direct release of NO and to the production of NO after first forming an epoxide structure. A previously characterized roaming-like path is not reported, being now replaced by the here*

described new roaming mechanism." Additionally, the next sentence has been added to the Figure 3 caption: "*On the right side, the here described new paths are depicted, while on the left side the path described in reference 15 are presented.*"

5- The referee states: "The authors presented the non-roaming reaction pathway on both the S0 and T1 surfaces starting from 3($n_B\pi^*$)CO in Figure 2 and the evolution of the S0 CASSCF(14,11) energy along the dynamics on the S0 state in Figure S6. On the one hand, it is recommended that the authors include information about the evolution of the T1 CASSCF(14,11) energy and describe related details, such as time step, based on the work [Nat. Chem., 14, 914-919]."

In the present contribution a non-roaming reaction pathway leading to NO_2 is presented which occurs entirely on the triplet manifold. A roaming reaction path leading to NO is also presented, which occurs initially on the triplet manifold, but then develops on the S_0 surface. We do not present here a non-roaming reaction on both the S_0 and T_1 surfaces starting from the $^3(n_B\pi^*)_{CO}$ geometry and Figure 2 represents only the roaming mechanism leading to NO. In Figure 2, $^3(n_B\pi^*)_{CO}$ is the final structure along the dynamics on the T_1 surface started from the $^3(n_B\pi^*)_{TS\text{-}dft}$ structure, and is the starting point of the performed dynamics on the S_0 surface leading to the nitrite isomer. We do not start any dynamics on the T_1 surface from the $^3(n_B\pi^*)_{CO}$ point.

Hovewer, as seems to be suggested by the referee, an analogous graph to the one reported in the original Figure S6 describing the evolution of the S_0 CASSCF(14,11) energy along the dynamics from $^3(n_B\pi^*)_{CO}$, is now presented in a new Figure S7, reporting the evolution of the T_1 CASSCF(14,11) energy along the dynamics from $^3(n_B\pi^*)_{TS\text{-}dft}$.

6- The referee states: "Although the title mentions "nitrobenzene photorelease mechanism of NO2and its relation to NO formation through a roaming mechanism", such relation is indeed lacking. Throughout the entire manuscript, there is a predominant focus on the non-roaming reaction pathway. Please reassess the significance of this work."

In the present contribution it is shown how along the photoinduced mechanism of formation of NO_2, the passage from T_1 to S_0 can instead lead to the formation of NO accordingly to a roaming mechanism, consequently linking the two processes. We thus disagree with the referee and we reaffirm that indeed one of the main focuses of our paper is the relation between the two photoinduced mechanisms.

7- The referee states: "... The authors should calculate CASPT2(16,13) energies for each statethroughout the entire scanning process, which may bring more intriguing results."

Such energies have been in fact calculated and reported in Figure S2 of the original contribution. However, for the sake of clarity, a table with all the energies has now been included as Table S1.

8- The referee states: "In fact, the 3(nBπ*) state passes from being the T4 at the Franck-Condonregion, to be the lowest triplet state, beside the σσ*, when the C-NO2 distance is equal to 2.15 Å." However, the result of T4 state is not clarified. Please cite the related reference or include further discussions."

In the reported sentence of the original text, we describe the fact that the triplet $^3(n_B\pi^*)$ state pass from being the T_4 state (i.e. the fourth lowest in energy triplet state) at the ground state minimum to

be the T_2 state when the C-NO_2 bond has been elongated to the value of 2.15 Å. This, as further discussed in the original text, proves that such a state is less destabilized than the other triplet states when the C-NO_2 bond is elongated.

9- The referee states: "The author mentioned “electron density and perform TDDFT computation”.

However, the manuscript lacks a clear description of this aspect."

The missing information is provided as Figure S5, now cited in the main text (page 4) as follows: "*From the analysis of the corresponding DFT electron density, orbitals, and performing a TDDFT computation, the T_1 state at the characterized TS geometry has indeed a marked $^3(n_B\pi^*)$ character (see Figure S5)*"

10- The referee states: "For the roaming reaction pathway, the authors should calculate quasi-classical dynamics or special post-TS dynamics, rather than CASSCF classical dynamics."

For nitrobenzene, whose electronic structure has been proven to be particularly challenging and to require the use of a large active space (see for example Phys. Chem. Chem. Phys., 2014, 16, 12393 and J. Phys. Chem. A 2021, 125, 9431), performing semi-classical dynamics accounting for nonadiabatic events and describing long-range motions (such as the one characterizing the roaming mechanism) would require a timescale of at least of 8.8 picosecond (the time scale of formation accordingly to Chem. Asian. J. 2006, 1 – 2, 56 – 63). This is simply not computationally affordable at present.

Minor issues

1- The referee states: "The authors used eV as the energy unit, which is seemingly popular in condensed-phase, but much less in molecular chemistry nor the previous roaming studies. This issue is not critical."

In order to favor the reading of the work, we have now introduced in the Supporting Information a new figure (Figure S9) that is equivalent to Figure 3 of the main text, but with all energies in kcal/mol.

Comments and changes after Referee 2 We

thank the referee for his/her helpful remarks.

Main concerns:

1- The referee states: "The opening paragraph needs to be rephrased in terms of the motivation forthe present work, the photochemistry of nitrobenzene is anything but prototypical, since there has been a lot of effort both experimentally and theoretically to address the complicated dynamics. Repetitive use of photophysical and photochemical words."

The term "prototypical" was here employed to indicate that nitrobenzene, being the smaller nitroaromatic compound, has been study as a reference molecule for the all family of nitroaromatic compounds. The referees objection is however understandable, and for that the opening paragraph has been rephrased as follows:

"*Nitrobenzene has recently attracted a lot of attention in the photophysical and photochemical community. This is easy understandable due, from one side, to its representative role in the nitroaromatic family, and, from the other side, to its peculiar photoinduced dynamics. Nitrobenzene photoresponse is in fact intrinsically key for basis science, displaying an unusual, for pure organic systems, ultrafast decay into the triplet manifold and a wide variety of photoinduced reactions. Moreover, its photochemistry has important applications in the energetic materials sector, in the study of urban atmospheric contaminants, and in the drug delivery sector.*"

2- The referee states: "... the motivation behind restricting the calculation between triplet statesmust be clearly be bought out."

We focused on triplet states mainly, because analogous mechanisms involving only the singlet manifold were not obtained, being characterized by significantly higher energies. In order to clarify this point, the following sentence can now been found on page 6 of the revised manuscript: "*Additionally, we attempted to evaluate whether a similar process could also occur entirely along the singlet manifold through the singlet $^1(n_B\pi^*)$ state, and obtained that much higher energies are required and that the S_0 always tends towards a bonded structure.* "

3- The referee states: "The author has shown the NO2 release by stretching the C-N bond from 1.47to 2.15 Å in the CASPT2 level. But nitro-nitrite photoisomerization through roaming dynamics occurs through C-N distance greater than 3 Å. Is the bond length stretching up to 2.15 Å enough to capture the actual dynamics? "

The study of the evolution of the energies of the low-lying triplet states from the value of 1.47 to the value of 2.15 Å has actually been performed in order to identify the state that favors (or, as it turns out to be the case, least disfavor) the elongation of the C-NO_2 bond. Once identified as the $^3(n_B\pi^*)$ state, the roaming process has been described to occur at larger C-NO_2 distances than 2.15 Å, being the representative structure of the passage from the NO_2 dissociation mechanism to the NO release roaming mechanism (i.e. the $^3(n_B\pi^*)_{CO}$ structure) characterized by a C-NO_2 distance of 3.227 Å.

4- The referee states: "... the author mentioned "At the C-NO2 distance of 2.0 Å, the statedescribing the σσ* excitation of the C-NO2 bond appears among the computed states, at an energy of 4.65 eV with respect to the ground state in its minimum. This indicates that the σσ* state is probably to high in energy to be directly involved in the NO2 photo release experimentally detected after 280-222 nm (4.43-5.58 eV) excitation." Probability of contribution from σσ*state at C-NO2 distance of 2.0 Å should be higher in between excitation range 4.43-5.58 eV. These sentences are confusing, needs better paraphrasing. Also "to high" should be "too high".".

The meaning of the mentioned paragraph was to stress that only after elongating the C-NO2 distance to as much as 0.53 Å the σσ* state makes its appearance among the lower states, which suggested that the σσ* state would be too high in energy at the Franck-Condon and nearby regions to be accessible after 280-222 nm excitation (4.43-5.58 eV). In order to better clarify this point, the last sentence (page 3) has been completed as follows:

"*This indicates that the σσ* state is probably too high in energy in the ground state minimum and thermally accessible structures to be directly involved in the NO_2 photorelease experimentally detected after 280-222 nm (4.43-5.58 eV) excitation.*"

5- The referee states: "...What is the magnitude of the singlet-triplet coupling constant at thiscrossing point?"

The value of the spin-orbit coupling at the characterized structures responsible for the T1 to S0 decay which consequently determine the passage from the NO2 photorelease mechanism to the NO roaming photorelease is lower than 1 cm^{-1} (0.75 cm^{-1} at the $^3(n_B\pi^*)_{CO}$ geometry). This is understandable as the T1 and S0 states have the same nature at this structure. The data is now reported in the revised version of the manuscript (page 5), where the original sentence "*Such a situation will in principle allow transfer of population from T_1 to S_0.*" has now been replaced by "*Such a situation will in principle allow transfer of population from T_1 to S_0, although the two display a low spin-orbit coupling of 0.75 cm^{-1} as a result of their common nature.*"

6- The referee states: "... Do the authors suggest that NO2 release is not a possible channel from theS0 state as however long the C-NO2 bond is elongated?"

Indeed our calculations point in such a direction. The S_0 state tends to evolve towards a bonded structure, either the nitro or the nitrite isomer.

Minor issues

7-11 The spotted typos have been corrected.

12- The referee states: "The term 'orientation of nitro group with respect to the aromatic ring isflipped' does not seem accurate here. The rotational reorientation indeed occurs leading to the oxygen atom being closer to the carbon, but the term 'flip' may not be the correct usage."

The term "flipped" has now been substituted by the term "inverted"

13- The referee states: "Page 2 Line 8: While discussing the work of Patwari, it may be ideal tomention that the work looks at the PES along the T1 surface and the dynamics on the triplet surface acts as a doorway between the roaming and non-roaming mechanisms."

Following the referee suggestion, the following sentence present in the original text: "*The recent work of Patwari support such a vision, using a simplistic model composed of two-dimensional potential energy surfaces (PESs) along the C-NO 2 and C-ONO coordinates.*" has now been replaced (page 2) by "*The recent work of Patwari based on the exploration of a two-dimensional model of the T_1 potential energy surface (PES) (the C-NO2 and C-ONO bond distances) support such a vision, concluding than the dynamics on the T_1 state acts as a doorway between the roaming and non-roaming mechanisms.*"

14- The spotted typo has been corrected.

Comments and changes after editorial comments

1- A TOC graphic has now been provided.

2- A brief, nonsentence description of the actual contents of each supporting information file is now provided.

3- The abstract has been shortend to 150 words

4- All references now use the JPCL formatting

5- URLs are not longer used

6- The SI pages are numbered in the following format: “S1, S2...”

7- We confirm that the reference citation included in some tables and figures pertains only to data and not the figure itself.

jz-2023-03457f.R2

Name: Peer Review Information for "A First Proposal on the Nitrobenzene Photorelease Mechanism of NO₂, and Its Relation to NO Formation Through a Roaming Mechanism"

Second Round of Reviewer Comments

Reviewer: 1

Comments to the Author

The authors addressed most of my comments to some extent. However, the most two important issues which mainly determine the quality of this roaming work were briefly handled. As this is a pure computational work, the quality of this current is still not enough for high-quality J. Phys. Chem. Lett. I can recommend this work after the authors seriously address these critical issues to enhance the quality.

1. The formatting of the figures in this revised manuscript, including TOC Graphic, Scheme 1, Figures 1 and S6-8 is still not up to the high standard of J. Phys. Chem. Lett. Please refer to previous studies for further revision [J. Phys. Chem. Lett. 2011, 2, 9, 934–938; J. Phys. Chem. Lett. 2020, 11, 4984–4989].

2. This work reveals the formation of C6H5 and NO2 products from ground-state C6H5ONO reactant through a singlet-triplet crossing point of phenyl and nitro groups. Is the new reaction pathway on the right side of Figure 3 a roaming reaction? Generally speaking, roaming is a reaction process in which a frustrated bond cleaves and a radical ‘roams’ around the remaining fragment over a long-range distance before eventually forming isomeric products. The potential surface in

the roaming region is often very flat and there are many recrossings of the dividing surface [Annu. Rev. Phys. Chem. 2020. 71:77–100]. The new 'roaming' pathway appears to deviate from that of formaldehyde ($H_2CO \rightarrow H + HCO \rightarrow H_2 + CO$), NO_3 and nitrobenzene. In other words, the singlet-triplet crossing point may be a roaming-mediated transition state, but not all of them exhibit the roaming properties. In addition, please provide more information about singlet-triplet crossing point, including Cartesian coordinates, the 'roaming' distances, bond angles or dihedral angles in Figure 3.

Reviewer: 2

Comments to the Author

The authors have made significant changes in the manuscript to address all the the comments made by two reviewers. This reviewer is satisfied with the changes made and recommends the manuscript for publication.

Author's Response to Peer Review Comments:

Valencia, February 13th, 2024

Dear Prof. Editor,

Please find attached the revised version of our paper entitled: "A first proposal on nitrobenzene photorelease mechanism of NO_2, and its relation to NO formation through roaming mechanism", by Angelo Giussani and Graham Worth.

The required three non-scientific changes have been now made in the new version of the manuscript. In addition, some smalls changes to the text for clarification and correction of some typos have been made. Finally, in partial fulfilment of the newer comments of referee 1, the coordinates of the singlet-triplet crossing regions have been added to the SI.

With our best regards,

Dr. Angelo Giussani
